# O05 - Once-daily tiotropium in adolescents with symptomatic asthma despite inhaled corticosteroid treatment: a dose-ranging study

**DOI:** 10.1186/2045-7022-4-S1-O5

**Published:** 2014-03-14

**Authors:** Christian Vogelberg, Michael Engel, Petra Moroni-Zentgraf, Migle Leonaviciute-Klimantaviciene, Ralf Sigmund, John Downie, Katja Nething, Viktorija Vevere, Mark Vandewalker

**Affiliations:** 1 University Hospital Carl Gustav Carus, Technical University of Dresden, Dresden, Germany; 2 Boehringer Ingelheim Pharma GmbH and Co. KG, Ingelheim am Rh, Germany; 3 Vilnius University Hospital, Vilnius, Lithuania; 4 Boehringer Ingelheim Pharma GmbH and Co. KG, Biberach an der, Germany; 5 Boehringer Ingelheim Pharmaceuticals Inc., Ridgefield, CT, USA; 6 Private Practice, Rēzekne, Latvia; 7 Clinical Research of the Ozarks, Columbia, MO, USA

## Background

Once-daily tiotropium is an effective and safe add-on bronchodilator for asthmatic adults who remain symptomatic despite inhaled corticosteroid (ICS) treatment in accordance with current international guidelines. Despite the wide range of available therapy options, many adolescents with asthma have disease that is sub-optimally controlled.

## Methods

This randomised, placebo-controlled, double-blind, incomplete crossover study (NCT01122680) evaluated the efficacy and safety of 5, 2.5 and 1.25 µg once-daily (evening) tiotropium (via Respimat^®^ Soft Mist™ Inhaler) versus placebo in three 4-week treatment periods in adolescents (aged 12-17 years) with symptomatic asthma despite medium-dose ICS. The primary efficacy end point was change in peak forced expiratory volume in 1 second within 3 hours post-dose (peak FEV_1(0-3h)_) assessed as a response (difference from baseline). Secondary end points included trough FEV_1_, FEV_1_ area under the curve (AUC)_(0-3h)_, peak expiratory flow (PEF_am/pm_) responses and Asthma Control Questionnaire (ACQ) score.

## Results

Of 139 enrolled patients, 105 were randomised to receive one of four treatment sequences. Peak FEV_1(0-3h)_ response was statistically significantly greater with 5 µg tiotropium than placebo (difference from placebo: 113±39 (SE) mL; p=0.0043). Trough FEV_1_ and FEV_1_ AUC_(0-3h)_ responses with 5 µg tiotropium were also significantly higher versus placebo (p<0.0001 and p=0.0001, respectively). A superior PEF_am_ and PEF_pm_ response was observed with 5 µg tiotropium over placebo. Although ACQ scores improved from baseline (2.091) with tiotropium (5 µg, 1.287; 2.5 µg, 1.366; 1.25 µg, 1.189), they also improved with placebo (1.371), which may be due to the short duration of this study. Safety profile was balanced across treatment groups, with the majority of adverse events being mild to moderate in severity and no dose-dependency observed.

## Conclusion

This first study of tiotropium as add-on to ICS in adolescents with symptomatic asthma demonstrates that 5 µg tiotropium is an effective and well-tolerated dose.

